# In silico screening of dicarboxylic acids for cocrystallization with phenylpiperazine derivatives based on both cocrystallization propensity and solubility advantage

**DOI:** 10.1007/s00894-017-3287-y

**Published:** 2017-03-27

**Authors:** Piotr Cysewski

**Affiliations:** Department of Physical Chemistry, Faculty of Pharmacy, Collegium Medicum of Bydgoszcz, Nicolaus Copernicus University in Toruń, Kurpińskiego 5, 85-950 Bydgoszcz, Poland

**Keywords:** Cocrystals, Solubility advantage, Mixing enthalpy, Dicarboxylic acids, Phenylpiperazine derivatives

## Abstract

In silico screening was performed to search for binary solids in which a phenylpiperazine-derivative drug was cocrystallized with a dicarboxylic acid. The phenylpiperazine derivative could be any of 61 such drugs, while the dicarboxylic acid could be any of nine such acids. The uniqueness of this approach was that two criteria had to be fulfilled simultaneously, namely a high propensity for cocrystallization and a sufficient solubility advantage. Using the mixing enthalpies of selected pairs of crystal formers with high affinities for one another permitted the classification of candidates with a high probability of cocrystallization. Further modeling of the solubility advantage allowed the identification of many binary solids that potentially exhibit significantly enhanced solubility in water. Based on the computed values for the mixing enthalpies and solubility advantage factors, it was concluded that dicarboxylic acids are both excellent coformers for cocrystallization with phenylpiperazines and very good solubility enhancers; indeed, the use of dicarboxylic acids as coformers would allow the degree of dissolution to be tuned for many of the studied drugs. The observed similarities of the cocrystallization landscapes of the studied drugs and excipients were also explored.

**Electronic supplementary material:**

The online version of this article (doi:10.1007/s00894-017-3287-y) contains supplementary material, which is available to authorized users.

## Introduction

Most organic compounds—including those of pharmacological interest—are poorly or very poorly soluble in water [1, 2], which complicates attempts to administer drugs directly and to make them bioavailable [3]. One way of overcoming this difficulty is to cocrystallize the active pharmaceutical ingredient (API; i.e., the drug) with a more soluble excipient [4]; in other words, cocrystallization can yield a solubility advantage. Given that this requires a good understanding of how the physicochemical properties of solids are altered by cocrystallization, it is clear to see why materials science [5] is playing an increasingly prominent role in drug development. Indeed, there are numerous examples of the advantages of drug cocrystallization, including improvements in the pharmacodynamic characteristics of APIs [6, 7]. However, while its advantageous effects on solubility [8], dissolution rate, and the dose–response relationship [9] as well as the possibility of synergistic effects caused by the cooperative action of several active substances [10] are all good reasons for attempting API cocrystallization [11], other physicochemical characteristics of drugs can also be improved by cocrystallization [12, 13]. For instance, cocrystallization has been employed to lower hygroscopicity [14, 15], increase physical or chemical stability [16, 17], modulate [18] and maintain [19] color, improve mechanical properties [14], control morphological characteristics, reduce the diversity of active forms of the substance [20], and to address issues relating to the patenting [21] of new solid forms of drugs [22]. All of these are important aspects of one of the last stages of drug development: the formulation of the most convenient solid form [23].

The wide variety of both active substances and coformers available on the market suggests that there are a huge number of possible combinations. However, not all substance pairs are miscible in the solid state, and predicting whether a solid dispersion takes the a form of physical mixture or an intermolecular compound is not a trivial task [24]. Moreover, successful cocrystallization does not guarantee that the new form will present a solubility advantage. Therefore, many different methods have been developed for the virtual screening of cocrystals of active pharmaceutical ingredients and to predict the solubility of cocrystals. In general, such screening methods can be divided into two broad classes. The first group of methods, often termed “ab initio” methods, directly model hypothetical solid structures while taking all of the properties of the crystal lattice into account [25]. This is accompanied by advanced and usually time-consuming quantum-chemical calculations, which consider many hypothetical crystal structures with various forms of symmetry. Some of the newest implementations of this approach have proven to be quite effective [26, 27]. The second group of methods ignore the characteristics of the crystal structure and focus on the properties of the interacting molecules derived directly from the structure of each coformer [24]. From the perspective of practicality [28], the first group of methods have limited applicability to cocrystal screening. Much more approximate methods often present surprisingly high efficiency despite the simplicity of the models used. For example, the statistical analysis of large populations of binary systems allows the classification of coformer properties that promote cocrystal formation [29, 30]. Furthermore, the role of intermolecular interactions in the formation of supramolecular patterns [31, 32] in single-component and multicomponent solids has been established in the literature through the introduction of the term “synthon” [33]. Alternatively, some methods that consider the electrostatic potential surface of the molecule have been used to identify the most likely contacts between components [24, 34, 35]. In particular, comparing values of the excess enthalpy of mixing (*H*_mix_) of coformers under supercooled conditions [36–38] has been found to be a very efficient way to screen for APIs that have a propensity to cocrystallize [25, 39–41]. Indeed, it has become possible to use this particular methodology [40] to rationalize the selection of pairs of coformers based on similarities in their affinities and cocrystallization landscapes. This approach takes into account linear relationships between the mixing enthalpies of components. As has been shown [40] for aromatic and heteroaromatic amides, the cocrystallization affinities of a set of chemical systems toward a group of coformers can be predicted after appropriate selection of a reference compound. This idea is in accord with chemical intuition, and can be very helpful when attempting to identify pairs with high probabilities of cocrystallization.

The work reported in the present paper focused on phenylpiperazine derivatives (PPDs), an important group of drugs that exhibit a variety of pharmacological activities. These compounds contain a phenylpiperazine skeleton formed by joining piperazine to a benzene moiety. Many representatives of this class have been successively marketed as valuable drugs. Probably the most well-known representative is itraconazole [42], which was first synthesized in 1984 and is a triazole antifungal agent. It has a very broad spectrum of activity against a variety of infections. Another well-known compound is ketoconazole [43], which is classified as both approved and investigational due to its broad spectrum of antifungal activities. It is used for long periods at high doses, especially in immunosuppressed patients, but also for the treatment of many systemic fungal infections such as chronic mucocutaneous candidiasis, oral thrush, blastomycosis, and paracoccidioidomycosis. However, many other phenylpiperazine-derivative drugs have been developed; a list of the most important is provided in Table 1.

**Table 1:** List of the most important phenylpiperazine derivatives considered in this paper

No.	Chemical name	Activity	Type	CAS	Code^a^
1	*N*-phenylpiperazine	–		92-54-6	
2	Antrafenine	Anti-inflammatory	Drug	55300-30-6	DB01419
3	Aripiprazole	Antipsychotic	Drug	129722-12-9	DB01238
4	Bifeprunox	Antipsychotic	Drug	350992-10-8	DB04888
5	Brexpiprazole	Antipsychotic	Drug	913611-97-9	DB09128
6	Cariprazine	Antipsychotic	Drug	839712-12-8	DB06016
7	Ciprofloxacin	Antibiotic	Drug	85721-33-1	DB00537
8	Dapiprazole	Alpha blocker	Drug	72822-12-9	DB00298
9	Dropropizine	Antitussive	Drug	17692-31-8	D07393
10	Elopiprazole	Antipsychotic	Drug	115464-77-2	
11	Eltoprazine	Serenic, antiaggressive	Drug	98206-09-8	
12	Etoperidone	Antidepressant	Drug	52942-31-1	
13	Itraconazole	Antifungal	Drug	84625-61-6	DB01167
14	Ketoconazole	Antifungal	Drug	79156-75-5	DB01026
15	Levodropropizine	Antitussive	Drug	99291-25-5	D08119
16	Levofloxacin	Antibiotic	Drug	100986-85-4	DB01137
17	Lubazodone	Antidepressant	Drug	161178-07-0	DB09196
18	Mepiprazole	Anxiolytic	Drug	20326-12-9	
19	Mianserin	Antidepressant	Drug	24219-97-4	DB06148
20	Moxifloxacin	Antibacterial	Drug	151096-09-2	DB00218
21	Naftopidil	Antihypertensive	Drug	57149-07-2	
22	Nefazodone	Antidepressant	Drug	83366-66-9	DB01149
23	Niaprazine	Hypnotic	Drug	27367-90-4	D07333
24	Oxypertine	Antipsychotic	Drug	153-87-7	D01219
25	Posaconazole	Antifungal	Drug	171228-49-2	DB01263
26	Tioperidone	Antipsychotic	Drug	52618-67-4	
27	Tolpiprazole	Tranquillizer, anxiolytic	Drug	20326-13-0	
28	Trazodone	Antidepressant	Drug	19794-93-5	DB00656
29	Umespirone	Antipsychotic	Drug	107736-98-1	
30	Urapidil	Antihypertensive	Drug	34661-75-1	
31	Vesnarinone	Cardiotonic	Drug	81840-15-5	
32	Acaprazine	Anxiolytic	Research	55485-20-6	
33	Batoprazine	Serenic, antiaggressive	Research	105685-11-8	
34	CHEMBL260870	Serotonergic	Research		
35	CHEMBL285066	Anxiolytic	Research		
36	CHEMBL534232	Serotonergic	Research	193611-72-2	
37	Enpiprazole	Anxiolytic	Research	31729-24-5	
38	Ensaculin	Nootropic	Research		
39	Flesinoxan	Antidepressant	Research	98206-10-1	D02568
40	Flibanserin	Aphrodisiac	Research	167933-07-5	D02577
41	Fluprazine	Serenic	Research	76716-60-4	
42	Lorpiprazole	Anxiolytic	Research	108785-69-9	
43	Naluzotan	Antidepressant	Research	740873-06-7	
44	Naphthylpiperazine	Serotonergic	Research	57536-86-4	
45	S-14506	Serotonergic	Research	135722-25-7	
46	S-14671	Serotonergic	Research	135722-27-9	
47	S-15535	Serotonergic	Research	146998-34-7	
48	SB-258585	Serotonergic	Research	209480-63-7	
49	SB-271046	Serotonergic	Research	CID5312149^b^	
50	SB-357134	Serotonergic	Research	CID6918649^b^	
51	SB-399885	Serotonergic	Research	402713-80-8	
52	Sonepiprazole	Dopaminergic	Research	170858-33-0	
53	Vortioxetine	Antidepressant	Research	508233-74-7	D10184
54	WAY-100135	Serotonergic	Research	133025-23-7	
55	WAY-100635	Serotonergic	Research	146714-97-8	
56	Zolertine	Antihypertensive	Research	4004-94-8	
57	1-(3-Chlorophenyl)piperazine	Serotonergic	Developed	6640-24-0	
58	1-(4-Chlorophenyl)piperazine	Serotonergic	Developed	38212-33-8	
59	2,3-Dichlorophenylpiperazine	Serotonergic	Developed	41202-77-1	
60	*Para*-fluorophenylpiperazine	Serotonergic	Developed	2252-63-3	
61	Trifluoromethylphenylpiperazine	Serotonergic	Developed	15532-75-9	

^a^DB = DrgBank, D = KEGG

^b^PubChem ID

The rest of this paper is organized as follows. First, an experimentally validated hypothesis regarding the transferability of cocrystallization landscapes is documented. Then, work done to screen a set of phenylpiperazine derivatives for good candidates for cocrystallization with dicarboxylic acids is reported (note that none of the derivatives had been cocrystallized previously). Finally, a subsequent investigation of the solubility advantage of each cocrystal highlighted by the screening process, based on in silico prediction, is discussed. To the author’s best knowledge, this is the first report of comprehensive screening involving the prediction of both drug cocrystallization ability and the solubility advantage of each new solid form identified.

## Methods

The following coformers were considered in this work: oxalic acid (0), malonic acid (1), succinic acid (2), glutaric acid (3), adipic acid (4), pimelic acid (5), suberic acid (6), azelaic acid (7), and sebacic acid (8), where each number in parentheses is the number of methylene groups in the chemical formula, i.e., *n* in HCOO(CH_2_)_*n*_COOH. All of these dicarboxylic acids (DCAs) appear in the EAFUS (Everything Added to Food in the United States) database [44] and the GRAS (Generally Recognized As Safe) list [45]. The affinities of these excipients for the APIs listed in Table 1 were quantified based on the estimated mixing enthalpy in the hypothetical supercooled state under ambient conditions. Enthalpy values were computed using the COSMOtherm software [46], utilizing the COSMO-RS (COnductor-like Screening MOdel for Real Solvents) approach [47, 48]. The cocrystallization propensities were estimated based on the correspondence between the miscibility in the solid state and that in liquids, as quantified by the mixing enthalpy:

1 $$ \Delta {H}_{12}^{mix}={x}_1{H}_{12}^1+{x}_2{H}_{12}^2-\left({x}_1{H}_1^1+{x}_2{H}_2^2\right), $$

where *x* denotes a mole fraction, superscripts indicate solvent types, and subscripts indicate solutes. This means that the excess enthalpy is obtained by subtracting the reference state values characterizing the pure components from the sum of the molar enthalpies of the components in the mixture. Technically, three calculations are necessary in which each of the pure components and the mixture are characterized by molar enthalpy values. The advanced level defined by the BP_TZVPD_FINE_C30_1601.ctd parameter set [46] was applied. The geometries of all compounds in both the gas and condensed phases were optimized using the BP-RI/TZVP scheme, which was followed by σ-profile computation by means of the BP-RI/TZVPD approach in Turbomole v7.0 [49] interfaced with TmoleX 4.2 [50].

In the second part of this investigation, the solubility advantage was estimated by computing the following measure:

2 $$ \mathrm{S}\mathrm{A}= \log \left(\frac{S_{\mathrm{CC}}}{S_{\mathrm{API}}}\right), $$

where *S*_cc_ denotes the solubility (in mol/L) of the cocrystal and *S*_API_ (in mol/L) is the drug solubility. The cocrystal solubility was computed via the salt solubility option in the COSMOtherm program, neglecting the contributions arising from the Gibbs free energy of fusion. When estimating API solubility, the iterative procedure was applied, and a QSPR model implemented in COSMOtherm was utilized for Δ*G*_fus_ estimation.

## Results and discussion

This section is divided into three parts, each addressing one of the major objectives of the work reported here. The main goal was to screen for the most promising drug–excipient pairs that not only had high probabilities of cocrystallization but also offered acceptable solubility advantages. This two-condition screening approach is very practical since it eliminates many of the cases that are not interesting from a practical pharmaceutical perspective. New solid forms of drugs are only useful if they offer an advantage over the single-component formulation. In order to successfully perform this final step, the newly proposed methodology [25, 40] of screening via analogy was validated and applied to the target group of drugs. The working paradigm for cocrystal screening is that miscibility in the solid state can be adequately predicted from the thermodynamics of the miscibility of liquids in the metastable supercooled state under ambient conditions. Unfortunately, as mentioned previously [25], detailed statistical analysis suggests that it is not possible to distinguish cocrystals from simple binary eutectics univocally. The number of misclassified cases heavily depends on the selected threshold value of *H*_mix_. Thus, an additional condition was suggested [40], which takes advantage of similarities in the cocrystallization landscapes of different substances that belong to the same class of compounds. Thus, the similarities of the cocrystallization landscapes of the nine dicarboxylic acids are documented here, as are the similarities of the cocrystallization landscapes of the phenylpiperazine derivatives.

### Transferability of the cocrystallization landscapes of DCAs

**Fig. 1:**
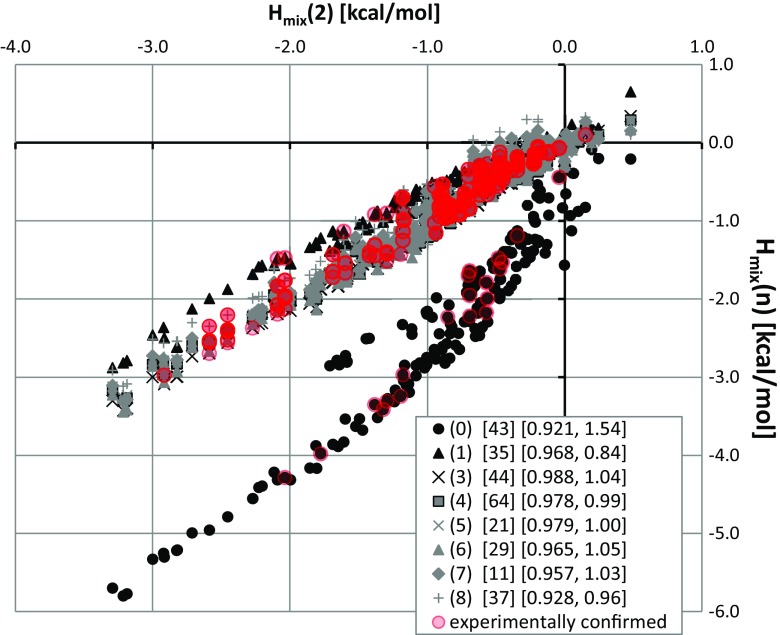
Distributions of *H*_mix_ values characterizing the affinities of the 175 coformers for the nine dicarboxylic acids (DCAs) with respect to the corresponding affinities of succinic acid (the reference DCA). The DCAs are labeled according to the number of methylene groups in the skeleton, i.e., by *n* in HCOO(CH_2_)_*n*_COOH: *(0)* oxalic acid, *(1)* malonic acid, *(2)* succinic acid, *(3)* glutaric acid, *(4)* adipic acid, *(5)* pimelic acid, *(6)* suberic acid, *(7)* azelaic acid, and *(8)* sebacic acid. The legend lists the number of experimentally derived cocrystals as well as the correlation coefficient *R* ^2^ and slope of the linear trend for each acid

In the first step, the cocrystallization landscapes of the dicarboxylic acids were characterized by listing all known binary solids that include these acids. This was done by searching within the Cambridge Structural Database (CSD) [51] and in the available literature [19, 52–59], and 374 cocrystals were obtained, all of which are documented in Table S1 of the “Electronic supplementary material” (ESM). Each of these bicomponent solids comprised one of the dicarboxylic acids interacting with one of 175 diverse coformers such as amino acids, drugs, amines, amides, phenols, other carboxylic acids, and many others. These cocrystals also varied in terms of the stoichiometry of the intermolecular complex. The majority (56%) of them exhibited a 1:1 ratio of components. Another 24% of the structures were characterized by a conformer:DCA stoichiometry of 2:1. A stoichiometry of 1:0.5 was found in 10% of the cocrystals. Other stoichiometries such as 0.5:1, 1:1.5, 2:3, and 3:1 were also observed, but they were quite rare. As mentioned in the “Methods” section, the affinities of the coformers were quantified based on values of the mixing enthalpy in the hypothetical supercooled metastable state under ambient conditions. Hence, *H*_mix_ values were computed for all 1575 binary mixtures defined as all possible combinations of the considered dicarboxylic acids with the compounds in the list of 175 coformers. The resulting values were plotted as a function of the *H*_mix_ of the selected reference compound. Succinic acid was chosen for this purpose as it is involved in as many as 90 cocrystals—no other dicarboxylic acid was used as frequently for binary solid synthesis. The resulting relationships (see Fig. 1) show interesting trends. The affinities of the dicarboxylic acids for of the considered coformers are quite similar to those characterizing succinic acid. Note that Fig. 1 presents two kinds of systems. The black and gray symbols are the possible combinations of coformers, including many that have not yet been synthesized. Overlaid on those data points are red symbols representing experimentally obtained cocrystals. The trends in both sets of data plotted suggest that the *H*_mix_ value distributions are similar for all DCAs except oxalic acid (this is understandable, as this compound is the most acidic of all the excipients studied here, and in many cases it is able to enforce salt formation with proton-accepting coformers). The main conclusion drawn from Fig. 1 is that knowledge of the cocrystallization abilities of succinic acid allows us to infer the cocrystallization characteristics of other DCAs—a conclusion supported by the relatively high correlation coefficients observed (*R* ^2^ > 0.9). This behavior has already been reported [40] for aromatic amides, when it was termed “the similarity of cocrystallization landscapes.” It appears that such behavior is exhibited many families of compounds. It is worth noting that this analogy is not based on a simple representation of the formal structure. For example, one of the strongest homosynthon systems is formed between two carboxylic groups. This structure is classified in graph theory as *R*_2_^2^(8), and is stabilized by two very strong hydrogen bonds. The contributions of these hydrogen bonds to the total stabilization energy of the crystal lattice can significantly exceed 50%; e.g., in crystals of aromatic carboxylic acids, the synthon stabilization energy exceeds the sum of the other kinds of intermolecular interactions that occur in the crystal lattice [60–62]. It is true that increasing the pressure can affect all types of interactions in a nonmonotonic manner [62], but the synthon stabilization energy still provides the dominant share of the total energy of the crystal. It is worth mentioning that linear trends [41, 63] between the stabilization energies of homo- and heterosynthons and the values of the Hammett constants *σ* describing the electrophilic and nucleophilic character of the substituents have been observed. However, the expectation that a similar relationship will also be observed for *H*_mix_ as a function of *σ* cannot be justified, as no such relationships have been found. Thus, while there is a simple relationship between the Hammett constants and the synthon stabilization energy [39], there is no similar relationship for *H*_mix_. This suggests that substituent effects make a nontrivial contribution to the total affinity of the coformers and it is not possible to infer the cocrystallization probability directly from the synthon energetics. This is clearly demonstrated by the statistical analysis of existing cocrystals, which show that the formation of heterosynthon patterns is much preferred to the formation of homosynthon patterns [64, 65]. For example, a homosynthon formed from two carboxyl groups is far less common than a heterosynthon generated from amide and carboxyl groups, despite the fact that the energy of a pair of carboxyl groups is generally a few to several kcal/mol higher than the energy of the amide and carboxyl pair [39, 62, 63]. In this context, while the linear trends observed in Fig. 1 correlate well with chemical intuition, they are not a trivial representation of the synthon energetics. This is also supported by the lack of simple rules governing cocrystallization within such a coherent class of compounds as aromatic carboxylic acids—not every pair of aromatic acids forms an intermolecular complex in the solid state, even though the stabilization energies of all such pairs are actually very similar [39, 41]. Admittedly, there have been suggestions of a correspondence between Hammett constant values and the cocrystallization abilities of two aromatic acids [66, 67], but such a relationship is only qualitative and not suitable for general cocrystal prognostics.

The predictive potential of the proposed analysis based on similarity can be demonstrated by inspecting particular cases. Table 2 presents a small portion of this dataset, which is included in its extended form in the ESM (Table S1). For example, seven cocrystals of isoniazid with DCAs are known. All of the acids show very strong affinity for this antibiotic which is used for the prevention and treatment of tuberculosis [68]. Therefore, it is very probable that it will also cocrystallize with oxalic and azelaic acids. Also, praziquantel (used as an anthelmintic agent for treating tapeworm and fluke infections [69]) will probably cocrystallize with suberic, azelaic, and sebacic acids. Using the contents of Table 2 (and the full dataset provided in the ESM), it is very easy to direct the synthesis of new solid forms. Note that not all of the drugs considered here will form cocrystals with dicarboxylic acids; for example, the affinities of almost all of the DCAs for paracetamol, stanozolol, etravirine, and cholesterin are so low that only oxalic acid forms cocrystals with those drugs. Also, this affinity-based approach does not always work. For instance, lamivudine—an antiretroviral medication that is used to prevent and treat HIV/AIDS—can be cocrystallized with pimelic acid (vis wax) despite the fact that the affinity of lamivudine toward DCAs is so low that its *H*_mix_ values are not sufficiently predictive. A statistical analysis [25] suggested that the precision with which pairs that form cocrystals can be distinguished from pairs that do not decreases as the value of *H*_mix_ increases.

**Table 2:** Examples of the transferability of cocrystallization landscapes characterizing the potential for the cocrystallization of various dicarboxylic acids with a few representative phenylpiperazine drugs

API	(0)	(1)	(2)	(3)	(4)	(5)	(6)	(7)	(8)
Isoniazid		FADGEY	FADGIC	FADGOI	FADGUO	FADHAV	SETRUG		SETROA
	+	(2:1)	(1:2)	(1:1)	(1:2)	(1:1)	(2:1)	+	(2:1)
	−2.61	−0.53	−0.89	−0.90	−0.79	−0.72	−0.73	−0.62	−0.53
Praziquantel	TELCOE	TELDEV	TELDAR	TELDIZ	TELCAQ	54]			
	(1:1)	(1:1)	(1:1)	(1:1)	(1:2)	(1:1)	+	+	+
	−3.31	−0.90	−1.30	−1.51	−1.42	−1.40	−1.55	−1.44	−1.29
Caffeine	GANXUP	GANYAW		EXUQUJ	CESKAN		[55]		
	(1:2)	(1:2)	+	(1:1)	(1:1)	+	(1:1)	+	+
	−2.47	−0.42	−0.63	−0.80	−0.69	−0.64	−0.73	−0.61	−0.45
Pyrazine	GUDSUV	GUDTAC	VAXWAU	GUDTOQ	GUDVAE				
	(1:1)	(1:1)	(1:1)	(1:1)	(1:1)	+	+	+	+
	−3.35	−0.92	−1.38	−1.45	−1.31	−1.29	−1.37	−1.27	−1.10
Carbamazepine	MOXWUS	MOXVUR	XOBCIB	MOXVOL	MOXVEB				
	(1:1)	(1:1)	(0.5:1)	(1:1)	(0.5:1)	+	+	+	+
	−1.48	−0.32	−0.48	−0.56	−0.52	−0.50	−0.54	−0.48	−0.43
Pyrazinamide		SIHRAE	LATTOR	SIHQOR	[17]				[17]
	+	(1:1)	(0.5:1)	(1:1)	(1:1)	+	+	+	(1:1)
	−2.25	−0.43	−0.70	−0.71	−0.60	−0.55	−0.55	−0.45	−0.34
Phenazine	XAPMIK	ZUPLEB	WOQBOT	WOQBUZ					
	(1:1)	(2:1)	(1:1)	(2:1)	+	+	+	+	+
	−3.24	−0.71	−1.19	−1.43	−1.34	−1.28	−1.44	−1.30	−1.17
Urotropine					MIPVEM	IJETOG		FITQII	EKECOM
	+	+	+	+	(1:1)	(1:1)	+	(1:1)	(1:1)
	−5.21	−2.61	−2.82	−2.99	−2.83	−2.78	−2.92	−2.78	−2.54
Theophylline	XEJWUF	XEJXAM		XEJXIU					
	(1:2)	(1:1)	+	(1:1)	+	+	+	+	+
	−1.99	−0.30	−0.47	−0.58	−0.48	−0.42	−0.48	−0.37	−0.26
Lamivudine						VISWAX			
	+	–	+	–	–	(1:2)	–	–	–
	−1.24	−0.17	−0.21	−0.17	−0.14	−0.05	0.01	0.09	0.09

The codes FADGEY, FADGIC, etc. relate to the record for this cocrystal in the Cambridge Structural Database (CSD). The data given for each cocrystal below the CSD code (if present; sometimes a relevant reference is cited instead, and sometimes there is no code nor reference for the cocrystal) are the stoichiometry of the cocrystals (if known; otherwise a “plus” symbol is shown) and the computed value of *H*_mix_ (in kcal/mol). The numbers heading the columns are consistent with the DCA numbering scheme used in Fig. 1

### Cocrystallization landscapes for phenylpiperazine derivatives

**Fig. 2:**
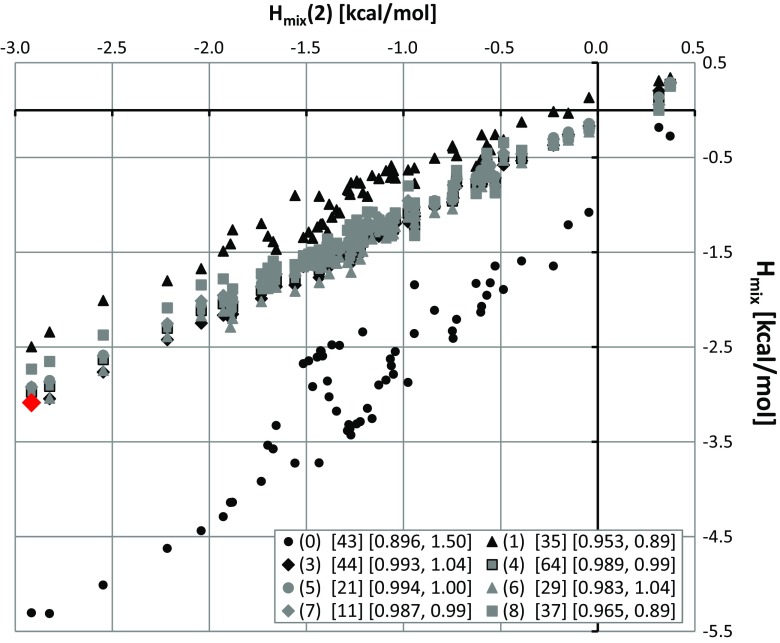
Distribution of *H*_mix_ values characterizing the affinities of 61 phenylpiperazine analogs for nine dicarboxylic acids. The notation is the same as that adopted in Fig. 1

There are only a few known cocrystals of PPDs with dicarboxylic acids. Indeed, succinic acid is involved in only two cocrystals. One is formed with ketoconazole and the second with itraconazole. The corresponding structures were deposited in the CSD under the codes YINWEZ and IKEQEU, respectively. Also, adipic acid was successfully cocrystallized with ketoconazole (YINWID). The other drugs presented in Table 1 have not been studied experimentally in terms of their cocrystallization potential. Thus, the results of the in silico screening presented in Fig. 2 are the only collection of potential cocrystals of phenylpiperazine drugs. Inspection of the plots suggests that many PPDs have high potential to cocrystallize with DCAs, and many binary solids could be synthesized. Hence, dicarboxylic acids are good choices for cocrystallization agents with phenylpiperazine derivatives, as the values of *H*_mix_ indicate strong affinities between such coformers in the majority of cases. Furthermore, many of the PPDs present the same mixing enthalpy distributions. However, not all PPDs would be expected to cocrystallize with DCAs. For example, the probability of successfully cocrystallizing an antipsychotic agent such as bifeprunox with DAs (except for oxalic acid) is low. However, such cases are quite rare: among the 549 binary systems formed between 9 DCAs and 61 PPDs, only 25 do not fulfill the miscibility criterion (*H*_mix_ < −0.17 [25]). Some results of the cocrystal screening performed in this work for three selected PPDs are collected in Table 3. The provided *H*_mix_ values strongly suggest that all of the DCAs are able to form intermolecular complexes with all of these drugs. As expected, the highest affinities are obtained for oxalic acid, but even the least acidic coformer (8) would be expected to be miscible in the solid state with these PPDs. The full list of results from the in silico screening performed here is provided in Table S2 of the ESM.

**Table 3:** Cocrystal screening results for three selected phenylpiperazines

	Ketoconazole	Dapiprazole	Itraconazole
(0)	−5.31	−5.31	−3.31
(1)	−2.50	−2.34	−0.75
(2)	−2.91^a^	−2.82	−1.24^b^
(3)	−3.09	−3.05	−1.43
(4)	−2.97^c^	−2.92	−1.34
(5)	−2.92	−2.85	−1.31
(6)	−3.07	−3.04	−1.44
(7)	−2.92	−2.87	−1.32
(8)	−2.73	−2.65	−1.16

The full list is provided in Table S2 of the ESM

^a^YINWEZ

^b^IKEQEU

^c^YINWID

### Potential solubility advantage of the cocrystallization of PPDs with DCAs

**Fig. 3:**
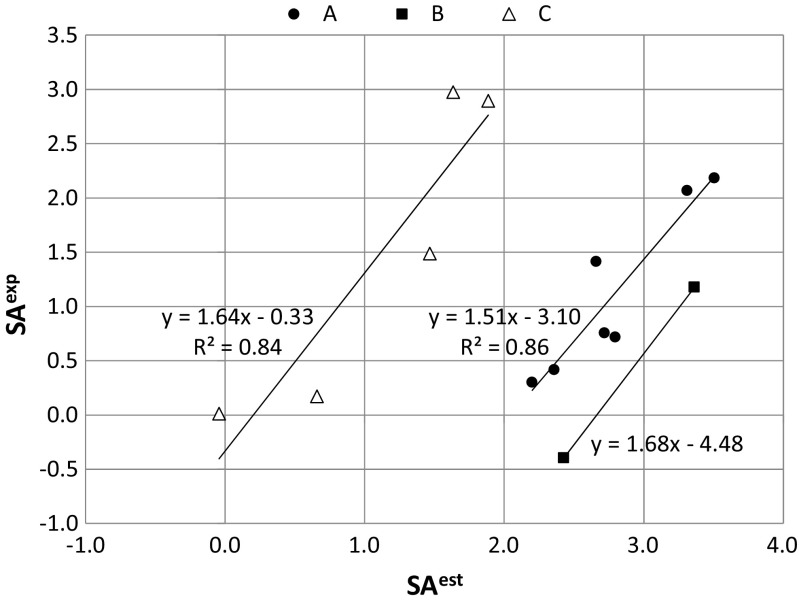
Correlations between the experimentally determined and the estimated solubility advantage values in water for three sets of data: *A* carbamazepine with saccharin, nicotinamide, succinic acid, malonic acid, oxalic acid, salicylic acid, or glutaric acid [6]; *B* theophylline with nicotinamide or salicylic acid [70]; *C* caffeine with malonic acid, glutaric acid, maleic acid, salicylic acid, or 1-hydroxy-2-naphthoic acid [71]

While knowledge of the cocrystallization probabilities of a series of PPDs is useful and interesting, it is not sufficient on its own. The synthesis of new binary solids of all APIs and drugs is a tedious and impractical path to generating new drug formulations. Since the solubility advantage of cocrystallization is very important, it is both necessary and interesting to predict the potential benefits of synthesizing new solid phases. This is why the screening process was also extended to include this important feature of new supramolecular systems of PPDs with DCAs. Since predicting the solubilities of drug-like substances is not a trivial task, and estimating cocrystal solubilities is even more problematic, it would be useful to perform some preliminary tests of the effectiveness of theoretical analysis. Thus, before actually screening for solubility advantage, it is necessary to validate this screening process using experimental data. In this work, an approach involving the estimation of the solubility advantage index was used, as described in the “Methods” section (see Eq. 2). Figure 3 presents plots of the experimentally measured solubility advantage for three sets of cocrystals in aqueous solution. Although the computed cocrystal and drug solubilities exhibit significant discrepancies from the available experimental data in terms of absolute values, the computed and experimental data do present similar trends. Thus, the computed values do appear to be useful despite the fact that they generally offer only a qualitative description of solubility trends. The trends presented in Fig. 3 indicate that higher predicted solubility advantage factors (SA^est^) are associated with higher experimentally observed increases in solubility after successful cocrystallization (SA^exp^). This qualitative trend could prove very useful when attempting to direct the synthesis of new cocrystals for further experimental verification. Thus, as a rule of thumb, it was assumed in this work that a sufficient solubility advantage can be expected if SA^est^ > 4, since this led to a significant gain in cocrystal water solubility (SA^exp^ > 1).

**Fig. 4:**
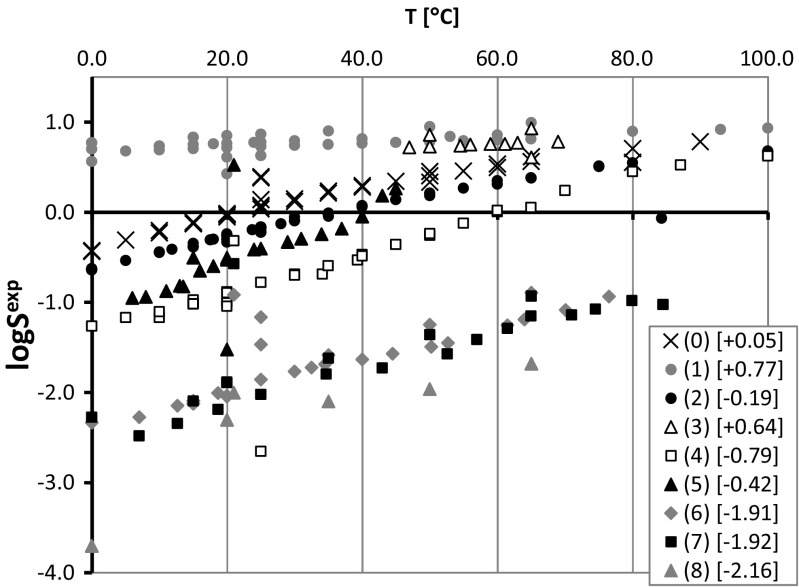
Experimental water solubilities [72] of dicarboxylic acids. Interpolated log*S* values for *T* = 25 °C are provided in the legend

**Fig. 5:**
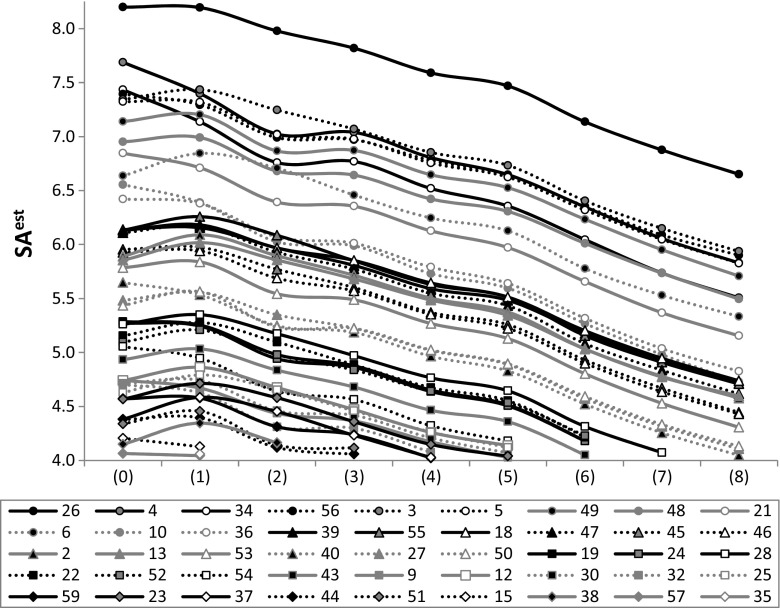
Predicted solubility advantage distributions for phenylpiperazine drugs after cocrystallization with dicarboxylic acids. The PPDs are labeled in the same manner as in Table 1

The utilization of dicarboxylic acids as potential solubility enhancers can be justified by the diversity of their water solubilities. Based on the data provided in Fig. 4, it is possible to divide DCAs into three classes. The first comprises the most soluble compounds (including oxalic, malonic, and glutaric acids), for which log*S* is positive at 25 °C. Modest solubility is expected with succinic, adipic, and pimelic acids, for which log*S* is negative but higher than −1. The other DCAs can be considered to exhibit low solubilities, with log*S* being less than −1. This high diversity of DCA solubilities in water is actually rather fortunate, as it offers the potential to tune the solubilities of the resulting binary solids within a wide possible solubility range. Unfortunately, there are no experimental data on the solubilities of either PPDs or their cocrystals, so theoretically derived values are the only data available. As expected, the predicted values of log*S* for the PPDs suggest that their water solubilities are very poor. For example, the most water soluble of the PPD drugs ciprofloxacin and fluprazine are still only barely soluble in water, since their estimated log*S* values are −4.4 and −4.3, respectively. The other phenypiperazine derivatives are even less soluble, and the least soluble (tioperidone and antrafenine) are characterized by ultralow values of log*S* = −12.2 and −11.6, respectively. These data confirm that it is worth studying the cocrystallization of PPDs as a means to increase their bioavailability. The computed solubility advantage factors suggest that DCAs are potentially important water-solubility enhancers for the PPD cocrystals considered here. In Fig. 5, the data fulfilling SA^est^ > 4 are plotted for intermolecular 1:1 complexes of PPDs with the nine dicarboxylic acids. 2:1 and 1:2 complexes were also considered, but those data did not change the general conclusions of the plot focusing on the 1:1 complexes. According to chemical intuition, the solubility of a cocrystal will depend on the characteristics of the dicarboxylic acid present. Indeed, a monotonic decrease in solubility advantage factors was observed to occur as the number of methylene groups in the DCA skeleton increased. The highest solubility gain was obtained for tioperidone (26). A positive effect of cocrystallization on water solubility can also be expected for many other PPDs, although not all—the solubility gain may be negligible in some cases. However, the data shown in Fig. 5 suggest that 45 of them are worth investigating experimentally.

## Conclusions

The in silico screening of both cocrystallization propensity and solubility advantage performed in the present work appears to provide valuable information enabling the rational planning of experiments. Based on these data, the synthesis of new solid forms of phenylpiperazine-derivative drugs can be effectively directed to maximize the pharmaceutically relevant benefits of those drugs. The proposed combined screening approach not only highlights pairs of coformers with high probabilities of cocrystallization but it also enables binary solids that will not provide a sufficient improvement in water solubility over that of the drug itself to be excluded. It is worth mentioning that due to a lack of Gibbs free energy of fusion values for the cocrystals, it is impossible to compute the absolute values of the cocrystal solubilities. This can be overcome by making use of some experimentally measured solubilities of some training cocrystals. Due to the existence of linear relationships between estimated solubilities and experimental solubility advantage values, in silico screening can be a very valuable tool when planning new experiments. Unfortunately, this did not apply to the phenylpiperazine drugs studied here, as the relevant experimental data are not available. However, even in such cases, developing a theoretical model is a good way to obtain very pragmatic guidance, as the model can yield a list of coformers with both the highest probabilities of cocrystallization and sufficient solubility advantage values. An arbitrary threshold is applied to guide coformer selection in such models, rather than general rules relating to the physical properties of the studied cocrystals. This categorization criterion can easily be tailored to meet the requirements of a specific drug after obtaining solubility measurements for some representative cases. Hence, even though the performed computations are qualitative assessments, the rational reduction of potential candidates for cocrystallization is a valuable aid to the development of new forms of the drugs studied here. However, each step of the proposed procedure should be refined further. In particular, the proposed methodology for selecting coformers based on similar affinities rather than trivial synthon-reflected characteristics may be especially important, particularly when *H*_mix_ has low predictive power. It is obvious that it is not possible to use the mixing enthalpy to explain the cocrystallization of a system that contains very similar compounds and has *H*_mix_ > −0.17 kcal/mol, yet such systems do exist. In those cases, however, observed linear trends with respect to a selected reference compound can enhance the applicability of miscibility data. In silico screening using a combination of these two criteria appears to provide valuable information enabling the rational planning and direction of experimental searches for new solid forms of active pharmaceutical ingredients.

## Electronic supplementary material

Below is the link to the electronic supplementary material.

ESM 1 (DOCX 95 kb)
