# Supplementary material for: In silico screening of dicarboxylic acids for cocrystallization with phenylpiperazine derivatives based on both cocrystallization propensity and solubility advantage
Source: J Mol Model. 2017 Mar 27;23(4):136. doi: 10.1007/s00894-017-3287-y (PMC5368210; doi:10.1007/s00894-017-3287-y)
Supplement: Supplementary file 1 — (DOCX 95 kb) [file 894_2017_3287_MOESM1_ESM.docx]

**Supporting information**

**Applicability of dicarboxylic acids as coformers for cocrystallization advantage
of phenylpiperazine derivatives**

Piotr Cysewski

**List of tables**

**Table S1**

The whole list characterizing cocrystallization landscapes of dicarboxylic acids with all 175 coformers. For experimentally obtained cocrystals the source and stoichiometry of components are provided. The values of H_mix_ are expressed in kcal/mol. Gray color denotes cases, for which miscibility criterion is not met (H_mix_<-0.18 kcal/mol [1]). Sigh plus or minus denotes predicted here cocrystallization or non-miscibility in the solid state, respectively. The following dicarboxylic acids were taken into account, namely oxalic acid (0), malonic acid (1), succinic acid (2), glutaric acid (3), adipic acid (4), pimelic acid (5), suberic acid (6), azelaic acid (7) and sebacic acid (8), where number represent n in the chemical formula, HCOO(CH_2_)_n_COOH.

**Table S2**

Results of in silico screening of phenypiperazine derivatives cocrystallization with dicarboxylic acids. Values of H_mix_ are expressed in kcal/mol.

**Table S1.**

The whole list characterizing cocrystallization landscapes of dicarboxylic acids with all 175 coformers. For experimentally obtained cocrystals the source and stoichiometry of components are provided. The values of H_mix_ are expressed in kcal/mol. Gray color denotes cases, for which miscibility criterion is not met (H_mix_<-0.18 kcal/mol [1]). Sigh plus or minus denotes predicted here cocrystallization or non-miscibility in the solid state, respectively. The following dicarboxylic acids were taken into account, namely oxalic acid (0), malonic acid (1), succinic acid (2), glutaric acid (3), adipic acid (4), pimelic acid (5), suberic acid (6), azelaic acid (7) and sebacic acid (8), where number represent n in the chemical formula, HCOO(CH_2_)_n_COOH.

|  | (0) | (1) | (2) | (3) | (4) | (5) | (6) | (7) | (8) |
| --- | --- | --- | --- | --- | --- | --- | --- | --- | --- |
| 2-pyridone | QIMHOJ (1:2) -1.19 | XUNHET (1:2) -0.23 | NISTAK (1:2) -0.35 | XUNHIX (1:1) -0.36 | [9] (1:1) -0.30 | XUNHOD (1:2) -0.28 | [2] (1:1) -0.25 | XUNHUJ (1:1) -0.19 | [2] (1:1) -0.18 |
| isonicotinamide | ULAWAF (1:2) -2.00 | ULAWEJ (1:2) -0.27 | LUNNUD (1:2) -0.56 | ULAWOT (1:2) -0.56 | ULAWUZ (1:2) -0.50 | ISIJEA (1:1) -0.43 | ISIJIE (1:1) -0.41 | ISIJAW (1:1) -0.35 | + -0.31 |
| nicotinamide | + -2.05 | NUKXUN (1:2) -0.32 | DUZPAQ (1:2) -0.61 | NUKYEY (1:1) -0.60 | NUKYIC (1:1) -0.54 | NUKYUO (1:1) -0.47 | NUKZAV (1:1) -0.45 | NUKZID (1:1) -0.39 | NUKZOJ (1:2) -0.35 |
| 2,4,6-triaminopyrimidine | [3] (1:1) -2.97 | [3] (1:1) -0.90 | [3] (1:1) -1.18 | [3] (1:1) -1.25 | (1:1) -1.14 | + -0.99 | [3] (1:1) -0.98 | + -0.74 | [3] (1:1) -0.70 |
| urea | UROXAL (1:2) -1.62 | URMALN (1:1) -0.42 | UNIRIT (1:1) -0.47 | TONGOS (2:1) -0.42 | ERIWUY (1:2) -0.26 | EVETAB (1:2) -0.19 | QQQBKM (1:1) -0.13 | - 0.00 | - 0.14 |
| 4,4'-bipyridine | XEZDIQ (1:1) -4.28 | SOVBOU (1:1) -1.48 | GOKBAL (1:1) -2.04 | SOVDIQ (1:1) -2.13 | [3] (1:1) -1.99 | XOLHUC (1:1) -1.96 | + -2.07 | + -1.96 | IFABUN (1:1) -1.77 |
| isoniazid | + -2.61 | FADGEY (2:1) -0.53 | FADGIC (1:2) -0.89 | FADGOI (1:1) -0.90 | FADGUO (1:2) -0.79 | FADHAV (1:1) -0.72 | SETRUG (2:1) -0.73 | + -0.62 | SETROA (2:1) -0.53 |
| N-(Pyridin-2-yl)isonicotinamide | + -2.29 | + -0.25 | KAYGUO (1:1) -0.56 | KAYFOH (1:1) -0.67 | KAYGEY (1:1) -0.61 | KAYFUN (1:1) -0.54 | KAYGIC (1:1) -0.60 | KAYGOI (1:1) -0.54 | KAYGAU (1:1) -0.48 |
| trans-1,2-bis(4-pyridyl)ethane | + -4.79 | + -1.88 | TACCIL (1:1) -2.45 | COZYEW (1:1) -2.56 | TACCUX01 (1:1) -2.42 | TACDAE (1:1) -2.40 | TACBEG (1:1) -2.52 | TACBIK (1:1) -2.41 | TACBAC (1:1) -2.21 |
| praziquantel | TELCOE (1:1) -3.31 | TELDEV (1:1) -0.90 | TELDAR (1:1) -1.30 | TELDIZ (1:1) -1.51 | TELCAQ (1:2) -1.42 | [4] (1:1) -1.40 | + -1.55 | + -1.44 | + -1.29 |
| imidazolidin-2-one | UHADAJ (1:2) -1.53 | UHADOX (1:3) -0.37 | UHADEN (2:1) -0.46 | UHADUD (1:1) -0.43 | UHADIR (2:1) -0.33 | UHAFAL (1:1) -0.30 | + -0.26 | + -0.19 | - -0.11 |
| 1,1'-bis(pyridin-4-ylmethyl)-1H,1'H-2,2'-biimidazole | + -4.31 | DOTBAR (1:1) -1.48 | DOSZAO (1:1) -2.09 | DOSPAE (2:1) -2.20 | DOSZES (1:1) -2.08 | + -2.04 | DOSZIW (1:1) -2.14 | + -2.02 | DOSZOC (1:1) -1.84 |
| 4-bromobenzamide | + -0.99 | HIQMOL (0.5:1) -0.15 | HIQSIL (1:2) -0.22 | + -0.26 | HIQSEH (2:1) -0.26 | HIQSAD (2:1) -0.21 | HIQRUW (2:1) -0.17 | - -0.13 | HIQROQ (2:1) -0.15 |
| N-(pyridin-2-yl)nicotinamide | + -2.37 | KAYHID (1:1) -0.30 | KAYHOJ (1:1) -0.62 | + -0.73 | KAYHAV (1:1) -0.67 | KAYHUP (1:1) -0.60 | + -0.66 | KAYJAX (1:1) -0.60 | KAYHEZ (1:1) -0.55 |
| caffeine | GANXUP (1:2) -2.47 | GANYAW (1:2) -0.42 | + -0.63 | EXUQUJ (1:1) -0.80 | CESKAN (1:1) -0.69 | + -0.64 | [5] (1:1) -0.73 | + -0.61 | + -0.45 |
| pyrazine | GUDSUV (1:1) -3.35 | GUDTAC (1:1) -0.92 | VAXWAU (1:1) -1.38 | GUDTOQ (1:1) -1.45 | GUDVAE (1:1) -1.31 | + -1.29 | + -1.37 | + -1.27 | + -1.10 |
| carbamazepine | MOXWUS (1:1) -1.48 | MOXVUR (1:1) -0.32 | XOBCIB (0.5:1) -0.48 | MOXVOL (1:1) -0.56 | MOXVEB (0.5:1) -0.52 | + -0.50 | + -0.54 | + -0.48 | + -0.43 |
| pyrazinamide | + -2.25 | SIHRAE (1:1) -0.43 | LATTOR (0.5:1) -0.70 | SIHQOR (1:1) -0.71 | [6] (1:1) -0.60 | + -0.55 | + -0.55 | + -0.45 | [6] (1:1) -0.34 |
| 4,4'-trimethylenedipyridine | + -4.96 | + -1.99 | JAZBES (1:1) -2.59 | TACBUW (1:1) -2.69 | TACCAD (1:1) -2.56 | TACCEH (1:1) -2.54 | + -2.67 | + -2.56 | TACBOQ (1:1) -2.35 |
| N-(2-Pyridyl)acetamide | + -2.30 | + -0.38 | KEFBED (1:2) -0.73 | + -0.83 | + -0.77 | KEFBON (1:2) -0.72 | KEFBUT (1:2) -0.77 | KEFCAA (1:2) -0.71 | KEFCEE (1:2) -0.65 |
| pyrazin-2-amine | + -2.45 | + -0.52 | WEDKOH (1:0.5) -0.91 | WEDKUN (1:1) -0.94 | + -0.87 | WEDLAU (1:1) -0.82 | WEDLEY (1:1) -0.84 | + -0.73 | WEDLIC (1:1) -0.67 |
| 4-hydroxybenzamide | GERZUA (0.5:1) -0.45 | - -0.11 | GESBAJ (0.5:1) -0.04 | GESBIR (0.5:1) -0.07 | - -0.09 | GESCAK (1:0.5) -0.06 | - 0.00 | - 0.00 | - 0.00 |
| phenazine | XAPMIK (1:1) -3.24 | ZUPLEB (2:1) -0.71 | WOQBOT (1:1) -1.19 | WOQBUZ (2:1) -1.43 | + -1.34 | + -1.28 | + -1.44 | + -1.30 | + -1.17 |
| picolinamide | + -1.43 | HOGGUH (1:1) -0.34 | HOGFIU (1:2) -0.45 | TOMWOJ (1:1) -0.48 | HOGFUG (1:0.5) -0.42 | + -0.38 | + -0.38 | + -0.31 | + -0.25 |
| N,N'-octane-1,8-diyldiisonicotinamide | + -2.65 | + -0.43 | CIWFUL (1:1) -0.76 | + -0.93 | CIWGAS (1:1) -0.89 | + -0.83 | CIWGEW (1:1) -0.93 | + -0.86 | CIWGIA (1:1) -0.81 |
| N,N'-hexane-1,6-diyldinicotinamide | + -2.82 | + -0.53 | CIWGOG (1:1) -0.87 | + -1.01 | CIWGUM (1:1) -0.95 | + -0.89 | CIWHAT (1:1) -0.97 | + -0.90 | CIWHEX (1:1) -0.83 |
| N,N'-hexane-1,6-diyldi-isonicotinamide | + -2.76 | + -0.53 | CUJMIE (1:1) -0.83 | + -0.98 | CUJMOK (1:1) -0.92 | + -0.86 | CUJMUQ (1:1) -0.95 | + -0.87 | CUJNAX (1:1) -0.80 |
| 3,3'-azopyridine | + -3.86 | + -1.14 | XEPBEB (1:1) -1.69 | + -1.79 | XEPBIF (1:1) -1.65 | + -1.62 | XEPBOL (1:1) -1.73 | + -1.61 | XEPBUR (1:1) -1.43 |
| 1,1'-bis(pyridin-2-ylmethyl)-1H,1'H-2,2'-biimidazole | + -3.52 | + -0.88 | + -1.36 | + -1.61 | DOSPEI (1:1) -1.52 | DOSQAF (2:1) -1.46 | DOSPIM (1:1) -1.62 | + -1.47 | DOSPOS (1:1) -1.33 |
| 2-amino-6-methyl-1,3-benzothiazole | + -0.71 | - -0.09 | + -0.18 | + -0.28 | LEXQEM (1:2) -0.32 | + -0.30 | FUHGUL (1:2) -0.32 | LEXQIQ (1:1) -0.27 | DUCXOP (1:2) -0.31 |
| urotropine | + -5.21 | + -2.61 | + -2.82 | + -2.99 | MIPVEM (1:1) -2.83 | IJETOG (1:1) -2.78 | + -2.92 | FITQII (1:1) -2.78 | EKECOM (1:1) -2.54 |
| benzoguanamine | [2] (2:1) -1.64 | + -0.52 | [2] (1:2) -0.69 | + -0.84 | [2] (1:2) -0.84 | + -0.78 | + -0.81 | + -0.66 | + -0.67 |
| N,N'-bis(2-pyridyl)-1,3-diaminobenzene | GABZEP (1:1) -2.23 | + -0.58 | GABZUF (1:1) -0.85 | + -0.87 | + -0.88 | + -0.79 | + -0.80 | + -0.77 | GABZIT (1:1) -0.83 |
| 2,4-diamino-6-phenyl-1,3,5-triazine | NIJGOE (0.5:1) -1.70 | + -0.52 | NIJGIY (0.5:1) -0.70 | + -0.85 | NIJHEV (1:0.5) -0.85 | + -0.79 | + -0.82 | + -0.67 | + -0.67 |
| theophylline | XEJWUF (1:2) -1.99 | XEJXAM (1:1) -0.30 | + -0.47 | XEJXIU (1:1) -0.58 | + -0.48 | + -0.42 | + -0.48 | + -0.37 | + -0.26 |
| 2-aminopyrimidine | + -2.65 | [5] (1:1) -0.56 | SERMOR (1:1) -0.95 | JOYJAK (1:1) -1.03 | + -0.94 | + -0.88 | + -0.93 | + -0.80 | + -0.71 |
| 1,3,4-thiadiazol-2-amine | + -1.75 | + -0.23 | HOQGIF (1:2) -0.47 | HOQGOL (1:1) -0.46 | HOQGUR (1:1) -0.38 | + -0.32 | + -0.29 | - -0.17 | - -0.12 |
| 5-bromo-2-propioamidopyridine | + -1.39 | - -0.01 | LADCOK (1:2) -0.23 | + -0.32 | + -0.31 | + -0.24 | LADCEA (2:1) -0.27 | + -0.24 | LADCUQ (2:1) -0.25 |
| 4-nitrobenzamide | + -0.93 | - -0.04 | PILKOM (0.5:1) -0.12 | YIWYAG (1:2) -0.14 | PILLAZ (1:0.5) -0.12 | - -0.07 | - -0.02 | - 0.03 | - 0.04 |
| 3-(4-picolinylamino)cyclohex-2-enone | + -3.54 | + -1.31 | QIPNAE (1:2) -1.60 | QIPNEI (1:1) -1.67 | QIPMUX (1:2) -1.54 | + -1.53 | + -1.56 | + -1.49 | + -1.38 |
| 4-(2-(3-thienyl)vinyl)pyridine | + -2.50 | + -1.09 | ROJBOJ (2:1) -1.42 | ROJCAW (1:2) -1.45 | ROJCEA (1:1) -1.40 | + -1.38 | + -1.44 | + -1.39 | + -1.31 |
| temozolomide | ACESEI (0.5:1) -2.18 | + -0.47 | ACESUY (0.5:1) -0.57 | + -0.56 | + -0.42 | + -0.32 | + -0.29 | - -0.15 | - -0.03 |
| 4-chlorobenzamide | GENLIV (1:2) -0.95 | - -0.11 | + -0.23 | + -0.30 | + -0.32 | + -0.29 | + -0.27 | + -0.25 | GENLOB (2:1) -0.27 |
| 4-Methylbenzamide | GENLUH (1:2) -1.20 | + -0.20 | + -0.34 | + -0.40 | + -0.39 | + -0.37 | + -0.35 | + -0.33 | GENMAO (2:1) -0.34 |
| theobromine | GORGUR (1:2) -2.36 | HIJYEF (1:1) -0.40 | + -0.61 | + -0.70 | + -0.58 | + -0.52 | + -0.55 | + -0.43 | + -0.30 |
| N-methyl-urea | MUROXA (2:1) -1.79 | + -0.48 | SUDDEC (2:1) -0.57 | + -0.56 | + -0.44 | + -0.39 | + -0.36 | + -0.27 | - -0.17 |
| 4-(1H-pyrazol-1-ylmethyl)benzamide | OCIPOG (0.5:1) -2.22 | + -0.39 | OCIPUM (0.5:1) -0.69 | + -0.77 | + -0.72 | + -0.66 | + -0.67 | + -0.59 | + -0.55 |
| bis(4-pyridyl)diazene | UMINAF (1:1) -3.98 | + -1.23 | GALBIF (1:1) -1.78 | + -1.88 | + -1.74 | + -1.71 | + -1.82 | + -1.71 | + -1.52 |
| 2,2'-bipyridyl-N,N'-dioxide | WOJHIM (1:1) -3.41 | + -1.02 | WOJHEI (1:1) -1.32 | + -1.46 | + -1.32 | + -1.26 | + -1.34 | + -1.19 | + -1.01 |
| N,N-dimethylurea | ZEFFEX (1:2) -1.96 | + -0.60 | SUCTAN (1:2) -0.69 | + -0.73 | + -0.65 | + -0.60 | + -0.59 | + -0.54 | + -0.49 |
| ketoprofen | + -0.83 | [7] (1:1) 0.10 | - -0.01 | [7] (1:1) -0.09 | - -0.08 | - -0.07 | - -0.12 | - -0.10 | - -0.07 |
| cytosine | + -1.41 | CUVDON (1:0.5) -0.36 | CUVDUT (0.5:1) -0.39 | + -0.30 | + -0.23 | - -0.14 | - -0.06 | - 0.06 | - 0.05 |
| 2,5-bis(4-pyridyl)-1,3,4-oxadiazole | + -3.83 | DAYZOT (1:1) -1.14 | GAWLOG (1:2) -1.61 | + -1.69 | + -1.53 | + -1.50 | + -1.58 | + -1.46 | + -1.25 |
| piracetam | + -2.65 | DIKDAD (1:1) -0.64 | DIKCIK (1:2) -0.89 | + -0.94 | + -0.82 | + -0.75 | + -0.76 | + -0.63 | + -0.53 |
| fluconazole | + -2.88 | MEWTAL (1:1) -0.62 | + -1.02 | UPOQIA (1:1) -1.07 | + -0.97 | + -0.91 | + -0.95 | + -0.83 | + -0.68 |
| benzimidazole | + -0.62 | MIZMUE (1:1) -0.13 | VARHUS (1:1) -0.18 | - -0.15 | - -0.16 | - -0.14 | - -0.13 | - -0.09 | - -0.10 |
| N-(4-((6-(4-(trifluoromethyl)phenyl)-4-pyrimidinyl)oxy)-1,3-benzothiazol-2-yl) acetamide | + -1.54 | MOXTOJ (0.5:1) -0.03 | + -0.29 | + -0.45 | MOXSAU (0.5:1) -0.45 | + -0.38 | + -0.43 | + -0.37 | + -0.38 |
| 4-amino-1,2,4-triazole | + -3.04 | + -0.81 | CIRXAD (1:1) -1.17 | + -1.14 | CIRXIL (1:2) -1.00 | + -0.91 | + -0.87 | + -0.71 | + -0.61 |
| 5-fluorocytosine | + -1.28 | + -0.25 | DILVAX (1:0.5) -0.19 | - -0.18 | DILVEB (0.5:1) -0.08 | - 0.03 | - 0.04 | - 0.16 | - 0.29 |
| 2-amino-4-ethyl-6-hydroxypyrimidine | + -1.25 | + -0.36 | [8] (1:1) -0.19 | + -0.18 | [8] (1:1) -0.06 | - -0.04 | - -0.01 | - 0.05 | - 0.27 |
| 1,3-bis((pyrid-2-ylamino)carbonyl)adamantine | + -2.77 | + -0.54 | RIZXAZ (1:1) -0.94 | + -1.12 | + -1.13 | + -1.04 | + -1.12 | + -1.09 | RIZWOM (1:1) -1.16 |
| nitazoxanide | + -0.83 | - 0.15 | SOCMII (0.5:1) 0.15 | [9] (1:2) 0.10 | - 0.16 | - 0.19 | - 0.18 | - 0.27 | - 0.32 |
| nicotinic acid hydrazide | + -2.57 | + -0.48 | UKOSIX (1:2) -0.86 | + -0.87 | UKOSAP (2:1) -0.78 | + -0.70 | + -0.70 | + -0.59 | + -0.53 |
| trans-1,2-bis(4-pyridyl)ethene | + -4.56 | + -1.69 | UMINOT (1:1) -2.27 | COZYIA (1:1) -2.37 | + -2.23 | + -2.21 | + -2.32 | + -2.21 | + -1.99 |
| ketoconazole | + -5.31 | + -2.50 | YINWEZ (1:1) -2.91 | + -3.09 | YINWID (1:1) -2.97 | + -2.92 | + -3.07 | + -2.92 | + -2.73 |
| meloxicam | + -0.94 | - 0.01 | - 0.04 | ENIBUZ (1:1) 0.01 | FAKJOS (1:0.5) 0.00 | - 0.05 | - 0.06 | - 0.06 | - 0.03 |
| 2-amino-4,6-dimethylpyrimidine | + -3.17 | + -0.83 | + -1.23 | NEBWIC (1:2) -1.43 | NEBWOI (2:1) -1.35 | + -1.28 | + -1.39 | + -1.24 | + -1.16 |
| 1,3-bis(((6-methylpyridy-2-yl)amino)carbonyl)-benzene | + -2.44 | + -0.47 | + -0.83 | YALKIF (1:1) -1.02 | + -1.03 | YALKEB (1:1) -0.93 | + -1.00 | + -0.96 | + -1.01 |
| 2-amino-4-hydroxy-6-methylpyrimidine | + -1.41 | + -0.43 | + -0.28 | ZUKXAE (1:1) -0.24 | ZUKXEI (1:2) -0.09 | - -0.06 | - -0.01 | - 0.08 | - 0.30 |
| N,N'-butane-1,4-diyldiisonicotinamide | + -2.78 | + -0.59 | + -0.87 | + -1.00 | CIWFIZ (1:1) -0.92 | + -0.86 | CIWFOF (1:1) -0.93 | + -0.85 | + -0.75 |
| 4-methyl-1,3-benzothiazol-2-amine | + -0.80 | - -0.16 | + -0.27 | + -0.43 | FICBIE (1:2) -0.48 | + -0.45 | FICBEA (1:2) -0.48 | + -0.41 | + -0.46 |
| sulfamethizole | + -1.26 | - -0.12 | + -0.19 | + -0.23 | OGUTOB (0.5:1) -0.21 | - -0.13 | OGUSUG (1:2) -0.08 | - 0.00 | - -0.01 |
| N'-(propan-2-ylidene)nicotinohydrazide | + -2.84 | + -0.81 | + -1.00 | + -1.07 | UKOSET (1:2) -1.00 | + -0.91 | + -0.95 | + -0.87 | UKOSOD (1:2) -0.84 |
| azopyridine | + -4.31 | + -1.55 | + -2.00 | + -2.15 | UMIMUY (1:1) -2.02 | + -1.95 | + -2.08 | + -1.93 | UMIMOS (1:1) -1.73 |
| 3,3',5,5'-tetramethyl-4,4'-bipyrazole | + -1.30 | - -0.10 | + -0.32 | + -0.33 | + -0.35 | + -0.32 | SOLJEK (0.5:1) -0.33 | + -0.30 | SOLJIO (1:0.5) -0.33 |
| 1,4-diazabicyclo[2.2.2]octane | + -5.78 | + -2.79 | + -3.19 | + -3.40 | + -3.27 | + -3.23 | UNEGAV (1:1) -3.42 | UNEGEZ (1:1) -3.29 | + -3.09 |
| efavirenz | (1:1) -0.09 | - 0.16 | - 0.20 | - 0.12 | - 0.04 | - 0.05 | - 0.05 | - 0.01 | - -0.06 |
| 1,3-benzothiazole | AYUMEN (0.5:1) -1.65 | + -0.30 | + -0.62 | + -0.69 | + -0.64 | + -0.62 | + -0.68 | + -0.62 | + -0.56 |
| l-phenyl-3,5-dimethylpyrazole | COGREV (1:2) -2.16 | + -0.67 | + -1.00 | + -1.15 | + -1.12 | + -1.08 | + -1.19 | + -1.12 | + -1.08 |
| 2,3,5,6-tetramethylpyrazine | COZYOG (1:1) -4.17 | + -1.32 | + -1.80 | + -2.10 | + -2.00 | + -1.93 | + -2.14 | + -1.98 | + -1.84 |
| furamide | FURAOX (1:2) -1.16 | + -0.21 | + -0.34 | + -0.38 | + -0.34 | + -0.31 | + -0.29 | + -0.23 | + -0.19 |
| N,N'-dimethylurea | KIDXEC (1:2) -1.93 | + -0.59 | + -0.68 | + -0.71 | + -0.64 | + -0.59 | + -0.58 | + -0.52 | + -0.47 |
| paracetamol | LUJTAM (1:1) -0.40 | - -0.01 | - 0.06 | - 0.05 | - 0.00 | - 0.03 | - 0.07 | - 0.03 | - 0.00 |
| acetamide | OXLACM (1:1) -1.40 | + -0.30 | + -0.44 | + -0.43 | + -0.36 | + -0.33 | + -0.30 | + -0.21 | - -0.17 |
| 4-cyanopyridine | PAVGOK (1:2) -2.24 | + -0.69 | + -0.96 | + -0.96 | + -0.87 | + -0.84 | + -0.86 | + -0.78 | + -0.69 |
| cholesterin | QEPFOI (0.5:1) -0.21 | - 0.65 | - 0.48 | - 0.34 | - 0.29 | - 0.27 | - 0.17 | - 0.15 | - 0.10 |
| trans-cinnamamide | RAHGEM (1:2) -1.14 | + -0.19 | + -0.34 | + -0.42 | + -0.41 | + -0.39 | + -0.40 | + -0.31 | + -0.33 |
| 3-(2-thienyl)acrylamide | RAHGOW (1:2) -1.19 | + -0.22 | + -0.37 | + -0.42 | + -0.40 | + -0.37 | + -0.36 | + -0.27 | + -0.28 |
| quinoxaline | RUXPUV (1:1) -3.22 | + -0.75 | + -1.21 | + -1.37 | + -1.26 | + -1.22 | + -1.34 | + -1.23 | + -1.08 |
| naphthyridine | RUXQAC (1:1) -3.89 | + -1.18 | + -1.65 | + -1.84 | + -1.72 | + -1.67 | + -1.80 | + -1.66 | + -1.50 |
| triphenylphosphine oxide | TPOXLC (1:2) -2.38 | + -0.90 | + -1.11 | + -1.29 | + -1.23 | + -1.22 | + -1.33 | + -1.25 | + -1.17 |
| 4,7-phenanthroline | WUKRAV (1:1) -4.17 | + -1.34 | + -1.85 | + -2.06 | + -1.93 | + -1.88 | + -2.02 | + -1.88 | + -1.70 |
| etiracetam | XOGPAM (1:1) -2.65 | + -0.60 | + -0.90 | + -0.99 | + -0.91 | + -0.86 | + -0.91 | + -0.83 | + -0.74 |
| levetiracetam | XOGPEQ (1:1) -2.44 | + -0.51 | + -0.77 | + -0.86 | + -0.78 | + -0.73 | + -0.77 | + -0.70 | + -0.64 |
| DL-histidine | + -2.12 | CAMWOD (1:1) -0.36 | + -0.64 | + -0.56 | + -0.45 | + -0.38 | + -0.34 | + -0.23 | - -0.14 |
| L-histidine | + -1.94 | CAMWUJ (1:1) -0.18 | + -0.47 | + -0.39 | + -0.30 | + -0.21 | - -0.16 | - -0.06 | - 0.01 |
| 1,1'-dibenzyl-1H,1'H-2,2'-biimidazole | + -1.54 | DOSQEJ (1:1) -0.26 | + -0.52 | + -0.64 | + -0.64 | + -0.59 | + -0.69 | + -0.63 | + -0.61 |
| 1-(5-(anthracen-10-yl)-3-(4-methoxyphenyl)-4,5-dihydropyrazol-1-yl)ethanone | + -2.17 | GEPTIG (1:1) -0.73 | + -1.01 | + -1.12 | + -1.09 | + -1.07 | + -1.15 | + -1.09 | + -1.04 |
| stanozolol | + -1.13 | VOPCOU (1:1) 0.23 | - 0.05 | - -0.04 | - -0.06 | - -0.04 | - -0.10 | - -0.10 | - -0.12 |
| 1,3-bis((benzimidazol-1-yl)methyl)benzene | + -4.41 | XAQPEL (1:1) -1.59 | + -2.23 | + -2.32 | + -2.21 | + -2.17 | + -2.26 | + -2.14 | + -1.97 |
| 1,4-bis(benzimidazol-1-ylmethyl)benzene | + -4.40 | XAQPIP (1:1) -1.57 | + -2.20 | + -2.31 | + -2.20 | + -2.16 | + -2.25 | + -2.13 | + -1.97 |
| drug 1a | + -2.18 | + -0.47 | [10] (1:1) -0.63 | + -0.70 | + -0.61 | + -0.55 | + -0.56 | + -0.46 | + -0.36 |
| phenanthridine | + -2.33 | + -0.84 | AXUFIJ (0.5:1) -1.18 | + -1.30 | + -1.26 | + -1.23 | + -1.32 | + -1.25 | + -1.19 |
| benzamide | + -1.20 | + -0.23 | BZASUC (1:2) -0.36 | + -0.41 | + -0.40 | + -0.36 | + -0.34 | + -0.31 | + -0.31 |
| 4-chloro-6-methoxypyrimidin-2-amine | + -1.24 | - -0.12 | EFAPUY (0.5:1) -0.27 | + -0.38 | + -0.37 | + -0.33 | + -0.37 | + -0.27 | + -0.26 |
| DL-valine | + -1.21 | + -0.18 | EWOZIZ (1:2) -0.30 | + -0.32 | + -0.30 | + -0.27 | + -0.30 | + -0.27 | + -0.24 |
| N'-hydroxypyridine-2-carboximidamide | + -2.48 | + -0.54 | FIDCIG (1:2) -0.66 | + -0.57 | + -0.43 | + -0.32 | + -0.29 | + -0.18 | - -0.09 |
| gabapentinlactam | + -1.61 | + -0.35 | GAXSIJ (0.5:1) -0.52 | + -0.60 | + -0.55 | + -0.56 | + -0.60 | + -0.56 | + -0.51 |
| itraconazole | + -3.31 | + -0.75 | IKEQEU (0.5:1) -1.24 | + -1.43 | + -1.34 | + -1.31 | + -1.44 | + -1.32 | + -1.16 |
| 1,2,4-triazole | + -1.99 | + -0.61 | IQIHEW (2:1) -0.93 | + -0.83 | + -0.74 | + -0.70 | + -0.68 | + -0.59 | + -0.52 |
| 1,3-bis(3-pyridyl)urea | + -2.32 | + -0.62 | JEDLAG (1:1) -0.83 | + -0.90 | + -0.81 | + -0.78 | + -0.85 | + -0.76 | + -0.58 |
| DL-proline | + -1.82 | + -0.49 | LABZUJ (0.5:1) -0.70 | + -0.70 | + -0.62 | + -0.59 | + -0.60 | + -0.53 | + -0.44 |
| 2-acetamido-5-bromopyridine | + -1.57 | - -0.08 | LADDIF (1:2) 0.00 | + -0.37 | + -0.34 | + -0.27 | + -0.29 | + -0.24 | + -0.22 |
| adefovir dipivoxil | + -3.19 | + -0.74 | LAPXEH (1:2) -1.13 | + -1.33 | + -1.24 | + -1.18 | + -1.30 | + -1.17 | + -1.04 |
| 2,2'-bipyridyl | + -1.30 | - 0.07 | LATJAT (1:1) -0.13 | + -0.27 | + -0.24 | + -0.19 | + -0.29 | + -0.21 | - -0.17 |
| 2,3-diphenylcycloprop-2-en-1-one | + -1.82 | + -0.42 | LATJEX (0.5:1) -0.69 | + -0.81 | + -0.76 | + -0.74 | + -0.81 | + -0.75 | + -0.68 |
| N-isopropylideneisonicotinohydrazide | + -3.10 | + -0.93 | LATKEY (0.5:1) -1.15 | + -1.24 | + -1.16 | + -1.08 | + -1.12 | + -1.04 | + -0.99 |
| N'-(butan-2-ylidene)isonicotinohydrazide | + -3.09 | + -0.90 | LATKIC (0.5:1) -1.14 | + -1.24 | + -1.16 | + -1.08 | + -1.15 | + -1.07 | + -1.00 |
| 1,4-bis((imidazol-1-yl)methyl)benzene | + -4.99 | + -2.13 | LATLEY (1:1) -2.71 | + -2.74 | + -2.60 | + -2.55 | + -2.62 | + -2.50 | + -2.30 |
| etoricoxib | + -3.28 | + -0.96 | MOGZEP (1:2) -1.29 | + -1.46 | + -1.36 | + -1.31 | + -1.41 | + -1.29 | + -1.15 |
| AMG517 | + -1.75 | - -0.03 | MOXSOI (0.5:1) -0.36 | - 0.00 | + -0.47 | + -0.40 | + -0.46 | + -0.39 | + -0.38 |
| trans-1,4-dithiane-1,4-dioxide | + -3.63 | + -1.23 | OGAHAF (1:1) -1.52 | + -1.58 | + -1.39 | + -1.33 | + -1.37 | + -1.21 | + -1.01 |
| (E)-2-methoxy-N-(2-(4-(3-methyl-4-(6-methylpyridin-3-yloxy)phenylamino)quinazolin-6-yl)ethenyl)acetamide | + -3.54 | + -1.05 | PEKQOM (1.5:1) -1.49 | + -1.67 | + -1.68 | + -1.57 | + -1.67 | + -1.63 | + -1.63 |
| 2,6-dimethylpyridine-N-oxide | + -2.20 | + -0.81 | QEVMEJ (1:1) -1.00 | + -1.11 | + -1.03 | + -1.02 | + -1.10 | + -1.03 | + -0.94 |
| 2,3-lutidine | + -2.80 | + -1.29 | RESGAY (1:2) -1.58 | + -1.70 | + -1.66 | + -1.63 | + -1.73 | + -1.67 | + -1.62 |
| 2,5-lutidine | + -2.84 | + -1.35 | RESGIG (1:2) -1.64 | + -1.74 | + -1.70 | + -1.68 | + -1.77 | + -1.72 | + -1.67 |
| 2,6-dimethylpyridine | + -2.81 | + -1.29 | RESGOM (1:2) -1.58 | + -1.74 | + -1.70 | + -1.66 | + -1.78 | + -1.71 | + -1.66 |
| 3,4-lutidine | + -2.86 | + -1.39 | RESHAZ (1:2) -1.71 | + -1.73 | + -1.67 | + -1.66 | + -1.73 | + -1.70 | + -1.63 |
| 3,5-dimethylpyridine | + -2.80 | + -1.32 | RESHIH (1:2) -1.65 | + -1.68 | + -1.63 | + -1.62 | + -1.69 | + -1.66 | + -1.59 |
| N-ethyl-N'-(1,3-thiazol-2-yl)isophthalamide | + -1.47 | - -0.09 | ROSTUQ (1:2) -0.25 | + -0.32 | - 0.00 | + -0.26 | + -0.25 | + -0.27 | + -0.30 |
| adenine | + -2.75 | + -0.67 | SEQVIV (1:1) -1.00 | + -1.01 | + -0.89 | + -0.79 | + -0.79 | + -0.63 | + -0.53 |
| bis(p-cyanophenyl)imidazolylmethane | + -1.41 | - -0.03 | SOSBAD (0.5:1) -0.14 | - -0.17 | - -0.13 | - -0.06 | - -0.07 | - 0.02 | - 0.04 |
| cis-1-((4-(1-imidazolylmethyl)cyclohexyl)methyl)imidazole | + -5.25 | + -2.36 | TAJVOP (1:1) -2.92 | + -2.96 | + -2.81 | + -2.78 | + -2.88 | + -2.76 | + -2.57 |
| pyrrolidin-2-one | + -1.77 | + -0.51 | UHACEM (2:1) -0.65 | + -0.68 | + -0.58 | + -0.57 | + -0.57 | + -0.52 | + -0.44 |
| 1-((1-benzimidazolyl)methyl)-4-(1-pyrazolylmethyl)benzene | + -3.88 | + -1.28 | VIGDEV (0.5:1) -1.81 | + -1.95 | + -1.85 | + -1.79 | + -1.90 | + -1.77 | + -1.62 |
| 2,2'-biimidazole | + -1.40 | + -0.24 | VORCOV (1:1) -0.41 | + -0.48 | + -0.47 | + -0.39 | + -0.40 | + -0.32 | + -0.31 |
| glycine | + -1.24 | + -0.27 | + -0.39 | AWIHOE (1:1) -0.29 | + -0.22 | + -0.18 | - -0.15 | - -0.09 | - -0.04 |
| nevirapine | + -2.29 | + -0.37 | + -0.69 | LATQEE (1:1) -0.87 | + -0.87 | + -0.77 | + -0.87 | + -0.80 | + -0.80 |
| N-(4-((6-(4-(trifluoromethyl)phenyl)pyrimidin-4-yl)oxy)-1,3-benzothiazol-2-yl) | + -1.84 | - -0.05 | + -0.39 | LEJLOD (1:1) -0.52 | + -0.49 | + -0.43 | + -0.50 | + -0.43 | + -0.39 |
| sulfathiazole | + -0.66 | - 0.00 | - 0.01 | LOFLUP (1:1) -0.04 | - -0.07 | - -0.01 | - 0.05 | - 0.11 | - 0.06 |
| 2-(pyridin-4-yl)-1H-benzimidazole | + -1.84 | + -0.37 | + -0.66 | OCEGEK (1:1) -0.75 | + -0.73 | + -0.66 | + -0.72 | + -0.67 | + -0.65 |
| 4-(2-(3,5-dimethoxyphenyl)vinyl)phenol | + -0.20 | - 0.18 | - 0.25 | OWAWAL (1:1) 0.15 | - 0.10 | - 0.09 | - 0.07 | - 0.04 | - 0.02 |
| acyclovir | + -2.38 | + -0.56 | + -0.67 | RIGDES (1:1) -0.53 | + -0.40 | + -0.29 | + -0.21 | - -0.11 | - 0.03 |
| 1,3-bis((pyrid-2-ylamino)carbonyl)adamantane | + -2.75 | + -0.61 | + -1.00 | RIZWEC (1:1) -1.16 | + -1.21 | + -1.10 | + -1.15 | + -1.17 | + -1.31 |
| mebendazole | + -1.87 | + -0.26 | + -0.51 | VEVPOD (1:1) -0.60 | + -0.55 | + -0.51 | + -0.56 | + -0.49 | + -0.43 |
| 6-(4-methoxyphenyl)-1,3,5-triazine-2,4-diamine | + -1.92 | + -0.51 | + -0.71 | + -0.86 | CEGGUS (0.5:1) -0.83 | + -0.76 | + -0.80 | + -0.63 | + -0.61 |
| clotrimazole | + -2.51 | + -1.12 | + -1.44 | + -1.47 | DUJXUD (1:2) -1.45 | + -1.44 | + -1.52 | + -1.48 | + -1.44 |
| 2,2'-(ethylenedioxy)diethylamine | + -5.70 | + -2.88 | + -3.29 | + -3.30 | GADQIL (1:1) -3.16 | + -3.12 | + -3.23 | + -3.11 | + -2.92 |
| N,N'-bis(6-methyl-2-pyridinyl)terephthalamide | + -2.74 | + -0.54 | + -0.94 | + -1.16 | JEWNUU (1:1) -1.16 | + -1.05 | + -1.15 | + -1.09 | + -1.12 |
| etravirine | + -0.88 | - 0.08 | - 0.10 | - 0.03 | LIKWIN (1:0.5) 0.01 | - 0.06 | - 0.08 | - 0.11 | - 0.09 |
| 3-methylpyridine | + -2.72 | + -1.26 | + -1.59 | + -1.62 | MOGVOV (1:2) -1.56 | + -1.55 | + -1.62 | + -1.58 | + -1.50 |
| 4-methylpyridine | + -2.79 | + -1.33 | + -1.66 | + -1.67 | MOGWEM (1:2) -1.61 | + -1.60 | + -1.67 | + -1.63 | + -1.55 |
| N-((4-pyridyl)methyl)acetamide | + -2.85 | + -0.87 | + -1.17 | + -1.20 | NEWSAK (1:2) -1.13 | + -1.08 | + -1.12 | + -1.05 | + -0.96 |
| 6'-methoxy-5-(4-(methylsulfonyl)phenyl)-3,3'-bipyridin-2-amine | + -2.82 | + -0.63 | + -1.05 | + -1.27 | PIWWEZ (1:2) -1.29 | + -1.17 | + -1.26 | + -1.21 | + -1.27 |
| N,N'-(ethane-1,2-diyldipyridine-6,2-diyl)diacetamide | + -2.59 | + -0.37 | + -0.79 | + -0.98 | QAGHEN (1:1) -0.95 | + -0.84 | + -0.94 | + -0.85 | + -0.81 |
| 2,4-diamino-6-methyl-1,3,5-triazine | + -2.85 | + -0.69 | + -1.08 | + -1.22 | ROGQUA (1:2) -1.14 | + -1.03 | + -1.07 | + -0.88 | + -0.83 |
| N,N'-(1,3-phenylenebis(methylene))bis(4,6-dimethylpyrimidin-2-amine) | + -2.98 | + -0.60 | + -1.08 | + -1.32 | TACTAU (1:1) -1.30 | + -1.21 | + -1.37 | + -1.24 | + -1.21 |
| 4,4'-ethene-1,2-diyldipyridine | + -4.56 | + -1.69 | + -2.27 | + -2.37 | UMINEJ (1:1) -2.23 | + -2.20 | + -2.32 | + -2.20 | + -1.99 |
| 5-chloro-2-pyridone | + -0.71 | - -0.03 | - -0.11 | - -0.14 | XASDEA (1:2) -0.12 | - -0.11 | - -0.09 | - -0.04 | - -0.08 |
| 2-amino-6-phenyl-4(1H)-pyrimidone | + -1.59 | + -0.47 | + -0.35 | + -0.35 | ZUKXUY (0.5:1) -0.25 | + -0.21 | + -0.19 | - -0.12 | - 0.03 |
| 4,4'-azopyridine | + -3.97 | + -1.22 | + -1.78 | + -1.88 | + -1.74 | + -1.71 | XEPCAY (1:1) -1.82 | + -1.70 | + -1.52 |
| blonanserin | + -2.45 | + -0.72 | + -1.11 | + -1.35 | + -1.35 | + -1.28 | XOJPAP (0.5:1) -1.47 | + -1.37 | + -1.36 |
| 2,5-bis(3-pyridyl)-1,3,4-oxadiazole | + -3.68 | + -1.02 | + -1.47 | + -1.56 | + -1.41 | + -1.37 | YECHIY (1:1) -1.45 | + -1.33 | + -1.12 |
| N,N'-bis(picolinoyl)hydrazine | + -1.90 | + -0.22 | + -0.41 | + -0.52 | + -0.43 | + -0.39 | YECHOE (1:1) -0.45 | + -0.35 | + -0.23 |
| 2-aminobenzothiazole | + -0.69 | - -0.13 | + -0.20 | + -0.29 | + -0.32 | + -0.30 | + -0.30 | LEXQOW (1:2) -0.24 | + -0.28 |
| 6-bromo-1,3-benzothiazol-2-amine | + -0.54 | - -0.16 | + -0.20 | + -0.30 | + -0.37 | + -0.35 | + -0.33 | + -0.26 | CIXBUI (1:2) -0.32 |
| 1,1'-methylenebis(1H-imidazole) | + -4.22 | + -1.57 | + -2.11 | + -2.10 | + -1.95 | + -1.89 | + -1.92 | + -1.79 | FAVFAL (1:1) -1.62 |
| 6-methoxy-1,3-benzothiazol-2-amine | + -1.02 | - -0.10 | + -0.21 | + -0.28 | + -0.29 | + -0.25 | + -0.26 | + -0.19 | FEXHOH (1:2) -0.20 |
| N,N'-bis(2-pyridyl)-1,4-diaminobenzene | + -2.36 | + -0.47 | + -0.82 | + -0.89 | + -0.89 | + -0.80 | + -0.83 | + -0.79 | GACBOC (1:1) -0.83 |
| N,N'-butylenebis(imidazole) | + -5.33 | + -2.46 | + -3.00 | + -3.00 | + -2.84 | + -2.79 | + -2.86 | + -2.73 | INEXIJ (1:1) -2.52 |
| 2,6-bis(((6-methylpyrid-2-yl)amino)carbonyl)naphthalene | + -2.74 | + -0.51 | + -0.96 | + -1.18 | + -1.19 | + -1.08 | + -1.18 | + -1.11 | JOHRAZ (1:1) -1.16 |
| 4-acetaminopyridine | + -2.08 | + -0.59 | + -0.84 | + -0.85 | + -0.80 | + -0.76 | + -0.78 | + -0.75 | NEWRUD (1:2) -0.69 |
| 1,3,5,7-tetra-azatricyclo(3.3.1.1^3,7^)decane | + -5.22 | + -2.62 | + -2.82 | + -3.00 | + -2.83 | + -2.78 | + -2.93 | + -2.78 | YEJKON (1:1) -2.54 |
| N-(4-bromo-2,3,5,6-tetrafluorophenyl)-2,3,5,6-tetrafluoro-4-iodobenzamide | + -5.80 | + -2.82 | + -3.21 | + -3.43 | + -3.30 | UNEFUO (1:1) -3.26 | + -3.45 | + -3.32 | + -3.12 |
| lamivudine | + -1.24 | - -0.17 | + -0.21 | - -0.17 | - -0.14 | VISWAX (1:2) -0.05 | - 0.01 | - 0.09 | - 0.09 |

**Table S2.**

Results of in silico screening of phenypiperazine derivatives cocrystallization with dicarboxylic acids. Values of H_mix_ are expressed in kcal/mol.

|  | (0) | (1) | (2) | (3) | (4) | (5) | (6) | (7) | (8) |
| --- | --- | --- | --- | --- | --- | --- | --- | --- | --- |
| Ketoconazole | + -5.31 | + -2.50 | YINWEZ -2.91 | + -3.09 | YINWID -2.97 | + -2.92 | + -3.07 | + -2.92 | + -2.73 |
| Dapiprazole | + -5.31 | + -2.34 | + -2.82 | + -3.05 | + -2.92 | + -2.85 | + -3.04 | + -2.87 | + -2.65 |
| Lorpiprazole | + -5.01 | + -2.01 | + -2.55 | + -2.77 | + -2.63 | + -2.59 | + -2.75 | + -2.59 | + -2.37 |
| Moxifloxacin | + -4.63 | + -1.80 | + -2.22 | + -2.42 | + -2.30 | + -2.26 | + -2.39 | + -2.25 | + -2.09 |
| Ciprofloxacin | + -4.44 | + -1.67 | + -2.04 | + -2.25 | + -2.12 | + -2.06 | + -2.17 | + -2.01 | + -1.84 |
| Levofloxacin | + -4.29 | + -1.49 | + -1.93 | + -2.17 | + -2.04 | + -1.98 | + -2.12 | + -1.96 | + -1.78 |
| Cariprazine | + -4.14 | + -1.41 | + -1.89 | + -2.20 | + -2.13 | + -2.06 | + -2.29 | + -2.12 | + -1.99 |
| Enpiprazole | + -4.14 | + -1.26 | + -1.88 | + -2.15 | + -2.08 | + -1.99 | + -2.20 | + -2.02 | + -1.89 |
| Ensaculin | + -3.92 | + -1.20 | + -1.73 | + -1.99 | + -1.90 | + -1.83 | + -2.02 | + -1.86 | + -1.73 |
| Lubazodone | + -3.54 | + -1.33 | + -1.70 | + -1.86 | + -1.78 | + -1.75 | + -1.88 | + -1.79 | + -1.67 |
| Batoprazine | + -3.58 | + -1.39 | + -1.67 | + -1.86 | + -1.77 | + -1.72 | + -1.84 | + -1.71 | + -1.60 |
| Eltoprazine | + -3.33 | + -1.47 | + -1.66 | + -1.86 | + -1.80 | + -1.72 | + -1.87 | + -1.73 | + -1.65 |
| Antrafenine | + -3.73 | + -0.90 | + -1.56 | + -1.85 | + -1.78 | + -1.70 | + -1.91 | + -1.75 | + -1.63 |
| N-Phenylpiperazine | + -2.68 | + -1.34 | + -1.52 | + -1.65 | + -1.60 | + -1.57 | + -1.66 | + -1.59 | + -1.54 |
| para-fluorophenylpiperazine | + -2.65 | + -1.29 | + -1.49 | + -1.63 | + -1.59 | + -1.55 | + -1.64 | + -1.57 | + -1.52 |
| SB-399885 | + -2.92 | + -1.35 | + -1.47 | + -1.62 | + -1.58 | + -1.54 | + -1.63 | + -1.55 | + -1.50 |
| Naphthylpiperazine | + -2.61 | + -1.23 | + -1.44 | + -1.60 | + -1.57 | + -1.54 | + -1.64 | + -1.57 | + -1.54 |
| Etoperidone | + -3.72 | + -0.91 | + -1.43 | + -1.77 | + -1.69 | + -1.59 | + -1.82 | + -1.63 | + -1.48 |
| 1-(3-Chlorophenyl)piperazin | + -2.54 | + -1.21 | + -1.43 | + -1.57 | + -1.54 | + -1.51 | + -1.60 | + -1.53 | + -1.49 |
| 1-(4-Chlorophenyl)piperazine | + -2.55 | + -1.20 | + -1.42 | + -1.57 | + -1.54 | + -1.50 | + -1.59 | + -1.52 | + -1.48 |
| Vortioxetine | + -2.60 | + -1.20 | + -1.42 | + -1.59 | + -1.57 | + -1.54 | + -1.66 | + -1.60 | + -1.58 |
| SB-357134 | + -2.86 | + -1.29 | + -1.39 | + -1.53 | + -1.49 | + -1.42 | + -1.49 | + -1.40 | + -1.36 |
| S-15535 | + -3.03 | + -0.99 | + -1.38 | + -1.66 | + -1.62 | + -1.51 | + -1.73 | + -1.56 | + -1.51 |
| Trifluoromethylphenylpiperazine | + -2.48 | + -1.13 | + -1.37 | + -1.52 | + -1.50 | + -1.47 | + -1.56 | + -1.49 | + -1.46 |
| Umespirone | + -3.18 | + -1.05 | + -1.34 | + -1.58 | + -1.53 | + -1.43 | + -1.62 | + -1.46 | + -1.40 |
| 2,3-Dichlorophenylpiperazine | + -2.48 | + -1.09 | + -1.33 | + -1.49 | + -1.48 | + -1.45 | + -1.55 | + -1.48 | + -1.45 |
| Trazodone | + -3.38 | + -0.84 | + -1.29 | + -1.58 | + -1.50 | + -1.41 | + -1.61 | + -1.42 | + -1.27 |
| Niaprazine | + -3.32 | + -0.85 | + -1.28 | + -1.55 | + -1.53 | + -1.40 | + -1.57 | + -1.43 | + -1.40 |
| CHEMBL285066 | + -3.36 | + -0.89 | + -1.27 | + -1.52 | + -1.41 | + -1.36 | + -1.51 | + -1.37 | + -1.21 |
| WAY-100635 | + -3.43 | + -0.77 | + -1.27 | + -1.61 | + -1.55 | + -1.46 | + -1.71 | + -1.52 | + -1.39 |
| Itraconazole | + -3.31 | + -0.75 | IKEQEU -1.24 | + -1.43 | + -1.34 | + -1.31 | + -1.44 | + -1.32 | + -1.16 |
| Nefazodone | + -3.29 | + -0.77 | + -1.22 | + -1.52 | + -1.45 | + -1.38 | + -1.57 | + -1.41 | + -1.26 |
| Mianserine | + -2.34 | + -0.87 | + -1.21 | + -1.41 | + -1.39 | + -1.35 | + -1.49 | + -1.41 | + -1.39 |
| Vesnarinone | + -3.15 | + -0.91 | + -1.19 | + -1.33 | + -1.23 | + -1.19 | + -1.27 | + -1.17 | + -1.08 |
| Posaconazole | + -3.26 | + -0.69 | + -1.16 | + -1.35 | + -1.26 | + -1.23 | + -1.36 | + -1.24 | + -1.08 |
| Sonepiprazole | + -2.90 | + -0.72 | + -1.13 | + -1.33 | + -1.30 | + -1.16 | + -1.27 | + -1.13 | + -1.16 |
| Aripiprazole | + -2.85 | + -0.64 | + -1.09 | + -1.31 | + -1.28 | + -1.20 | + -1.35 | + -1.23 | + -1.21 |
| Naluzotan | + -2.63 | + -0.71 | + -1.07 | + -1.23 | + -1.27 | + -1.13 | + -1.22 | + -1.17 | + -1.27 |
| S-14506 | + -2.70 | + -0.59 | + -1.06 | + -1.29 | + -1.32 | + -1.19 | + -1.33 | + -1.24 | + -1.29 |
| S-14671 | + -2.79 | + -0.64 | + -1.05 | + -1.28 | + -1.26 | + -1.15 | + -1.31 | + -1.18 | + -1.14 |
| Tolpiprazole | + -2.55 | + -0.72 | + -1.04 | + -1.20 | + -1.16 | + -1.10 | + -1.23 | + -1.13 | + -1.09 |
| Fluprazine | + -2.88 | + -0.63 | + -0.98 | + -1.18 | + -1.09 | + -1.01 | + -1.13 | + -0.96 | + -0.80 |
| Mepiprazole | + -2.36 | + -0.61 | + -0.94 | + -1.09 | + -1.06 | + -1.01 | + -1.12 | + -1.03 | + -0.98 |
| Elopiprazole | + -1.84 | + -0.77 | + -0.94 | + -1.12 | + -1.23 | + -1.09 | + -1.19 | + -1.15 | + -1.32 |
| CHEMBL534232 | + -2.11 | + -0.51 | + -0.84 | + -1.02 | + -0.99 | + -0.96 | + -1.08 | + -1.01 | + -0.97 |
| Tioperidone | + -2.33 | + -0.38 | + -0.75 | + -0.97 | + -0.96 | + -0.89 | + -1.04 | + -0.94 | + -0.92 |
| Flesinoxan | + -2.41 | + -0.40 | + -0.74 | + -0.93 | + -0.92 | + -0.80 | + -0.91 | + -0.79 | + -0.80 |
| Levodropropizine | + -2.21 | + -0.48 | + -0.73 | + -0.80 | + -0.72 | + -0.72 | + -0.77 | + -0.73 | + -0.64 |
| SB-271046 | + -1.83 | + -0.59 | + -0.63 | + -0.73 | + -0.80 | + -0.70 | + -0.72 | + -0.73 | + -0.88 |
| Urapidil | + -2.13 | + -0.53 | + -0.60 | + -0.71 | + -0.70 | + -0.64 | + -0.69 | + -0.68 | + -0.70 |
| Oxypertine | + -2.07 | + -0.26 | + -0.60 | + -0.78 | + -0.76 | + -0.67 | + -0.81 | + -0.68 | + -0.69 |
| Dropropizine | + -1.96 | + -0.37 | + -0.57 | + -0.65 | + -0.56 | + -0.55 | + -0.60 | + -0.56 | + -0.46 |
| SB-258585 | + -1.82 | + -0.42 | + -0.55 | + -0.66 | + -0.70 | + -0.59 | + -0.61 | + -0.59 | + -0.71 |
| WAY-100135 | + -1.65 | + -0.26 | + -0.53 | + -0.72 | + -0.76 | + -0.70 | + -0.82 | + -0.79 | + -0.88 |
| Zolertine | + -1.89 | + -0.32 | + -0.48 | + -0.58 | + -0.49 | + -0.47 | + -0.53 | + -0.45 | + -0.34 |
| Flibanserin | + -1.59 | - -0.13 | + -0.39 | + -0.53 | + -0.51 | + -0.45 | + -0.56 | + -0.47 | + -0.42 |
| Brexpiprazole | + -1.65 | - -0.02 | + -0.23 | + -0.37 | + -0.36 | + -0.29 | + -0.37 | + -0.31 | + -0.34 |
| Acaprazine | + -1.21 | - -0.03 | - -0.15 | + -0.26 | + -0.28 | + -0.23 | + -0.32 | + -0.28 | + -0.29 |
| Naftopidil | + -1.08 | - 0.13 | - -0.04 | - -0.17 | - -0.18 | - -0.14 | + -0.23 | + -0.19 | + -0.20 |
| CHEMBL260870 | + -0.19 | - 0.31 | - 0.32 | - 0.20 | - 0.13 | - 0.14 | - 0.09 | - 0.06 | - 0.00 |
| Bifeprunox | + -0.27 | - 0.34 | - 0.37 | - 0.29 | - 0.27 | - 0.29 | - 0.25 | - 0.28 | - 0.25 |

**References**

1. Cysewski P (2016) Efficacy of bi-component cocrystals and simple binary eutectics screening using heat of mixing estimated under super cooled conditions. J Mol Graph Model 68:23–28. doi: 10.1016/j.jmgm.2016.06.003

2. Jali BR, Baruah JB (2013) Cocrystals of 2,4-Diamino-6-phenyl-1,3,5-triazine with Dicarboxylic Acids. J Chem Crystallogr 43:531–537. doi: 10.1007/s10870-013-0453-7

3. Pedireddi VR, Chatterjee S, Ranganathan A, Rao CNR (1998) A study of supramolecular hydrogen bonded complexes formed by aliphatic dicarboxylic acids with azaaromatic donors. Tetrahedron 54:9457–9474. doi: 10.1016/S0040-4020(98)00574-2

4. Espinosa-Lara JC, Guzman-Villanueva D, Arenas-García JI, et al. (2013) Cocrystals of Active Pharmaceutical Ingredients—Praziquantel in Combination with Oxalic, Malonic, Succinic, Maleic, Fumaric, Glutaric, Adipic, And Pimelic Acids. Cryst Growth Des 13:169–185. doi: 10.1021/cg301314w

5. HIGUCHI T, LACH JL (1954) Investigation of some complexes formed in solution by caffeine. V. Interactions between caffeine and p-aminobenzoic acid, m-hydroxybenzoic acid, picric acid, o-phthalic acid, suberic acid, and valeric acid. J Am Pharm Assoc Am Pharm Assoc (Baltim) 43:524–7.

6. Wang J-R, Zhou C, Yu X, Mei X (2014) Stabilizing vitamin D(3) by conformationally selective co-crystallization. Chem Commun (Camb) 50:855–8. doi: 10.1039/c3cc47747a

7. Vangala VR, Chow PS, Tan RBH (2011) Characterization, physicochemical and photo-stability of a co-crystal involving an antibioticdrug, nitrofurantoin, and 4-hydroxybenzoic acid. CrystEngComm 13:759–762.

8. Liao R-F, Lauher JW, Fowler FW (1996) The application of the 2-amino-4-pyrimidones to supramolecular synthesis. Tetrahedron 52:3153–3162. doi: 10.1016/0040-4020(95)01101-3

9. Félix-Sonda BC, Rivera-Islas J, Herrera-Ruiz D, et al. (2014) Nitazoxanide Cocrystals in Combination with Succinic, Glutaric, and 2,5-Dihydroxybenzoic Acid. Cryst Growth Des 14:1086–1102. doi: 10.1021/cg4015916

10. Li Z, Yang B-S, Jiang M, et al. (2009) A Practical Solid Form Screen Approach To Identify a Pharmaceutical Glutaric Acid Cocrystal for Development. Org Process Res Dev 13:1307–1314. doi: 10.1021/op900137j
